# Violence Detection Using Spatiotemporal Features with 3D Convolutional Neural Network

**DOI:** 10.3390/s19112472

**Published:** 2019-05-30

**Authors:** Fath U Min Ullah, Amin Ullah, Khan Muhammad, Ijaz Ul Haq, Sung Wook Baik

**Affiliations:** 1 Intelligent Media Laboratory, Digital Contents Research Institute, Sejong University, Seoul 143-747, Korea; fath3797@gmail.com (F.U.M.U.); qamin3797@gmail.com (A.U.); hijaz3797@gmail.com (I.U.H.); 2 Department of Software, Sejong University, Seoul 143-747, Korea; Khan.muhammad@ieee.org

**Keywords:** abnormal activity, deep learning, 3D convolutional neural network, violence detection, surveillance cameras

## Abstract

The worldwide utilization of surveillance cameras in smart cities has enabled researchers to analyze a gigantic volume of data to ensure automatic monitoring. An enhanced security system in smart cities, schools, hospitals, and other surveillance domains is mandatory for the detection of violent or abnormal activities to avoid any casualties which could cause social, economic, and ecological damages. Automatic detection of violence for quick actions is very significant and can efficiently assist the concerned departments. In this paper, we propose a triple-staged end-to-end deep learning violence detection framework. First, persons are detected in the surveillance video stream using a light-weight convolutional neural network (CNN) model to reduce and overcome the voluminous processing of useless frames. Second, a sequence of 16 frames with detected persons is passed to 3D CNN, where the spatiotemporal features of these sequences are extracted and fed to the Softmax classifier. Furthermore, we optimized the 3D CNN model using an open visual inference and neural networks optimization toolkit developed by Intel, which converts the trained model into intermediate representation and adjusts it for optimal execution at the end platform for the final prediction of violent activity. After detection of a violent activity, an alert is transmitted to the nearest police station or security department to take prompt preventive actions. We found that our proposed method outperforms the existing state-of-the-art methods for different benchmark datasets.

## 1. Introduction

In the past decade, with the growth and advancements in the field of computer vision, an enormous amount of modern techniques has emerged and gained much attention among researchers due to their vast surveillance applications [1,2,3,4,5]. For instance, in 2017, about 954,261 CCTV cameras were installed in public in South Korea, which was an increase of 12.9% compared to the previous year [6]. The purpose of these cameras is to ensure security in public places. For this purpose, we focus on the detection of violence using these cameras. Violence is an abnormal behavior and an activity that involves some physical force to damage something, to kill or hurt a human or an animal; these actions can be identified through a smart surveillance system which could be used to prevent these events before further fatal accidents. One of the main functions of surveillance systems deployed on a large scale in different areas, such as schools, streets, parks, and medical centers, is to facilitate the authorities by alerting them to the violent activity. However, the response of human operators monitoring the surveillance footage is very slow, causing loss of human life and property; thus, there is a demand for an automated violence detection system [7]. Hence, this field of study is growing steadily and gaining interest in the computer vision society. Many techniques based on deep features [8,9,10] and handcrafted features have emerged.

### 1.1. Handcrafted Features-Based Approaches

In these approaches, certain methods are developed by the researchers. For instance, Datta et al. [11] used the trajectory of motion information and limb orientation of a person in the scene to detect violence. Similarly, Nguyen et al. [12] suggested the use of the hierarchical hidden Markov model (HHMM) to recognize violent activities. Their main contribution involves the utilization of a shared structure of HHMM for violence detection. Some of the researchers integrated audio and video modalities for the detection of violent activities. For instance, Mahadevan et al. [13] developed a system to recognize violent scenes via detecting blood and flames combined with the degree of motion and sound. A research work proposed by Hassner et al. [14] considered the flow vector magnitude represented by violent flow descriptors (ViF). Using a support vector machine (SVM), these ViF descriptors were then classified into violent and non-violent in crowd scenes. Furthermore, Huang et al. [15] presented a method for violent crowd behavior analysis by considering only the statistical properties of the optical flow field in video data. These properties were then classified into normal or abnormal activity classes using SVM. To detect and localize the violence in a surveillance video stream, Zhang et al. [16] presented a Gaussian model of optical flow for violent region extraction and used an orientation histogram of optical flow to distinguish the violent from non-violent class via linear SVM. Similar to this method, Gao et al. [17] proposed an oriented violent flow descriptor (OViF), which depicts both motion magnitude and orientation information.

### 1.2. Deep Learning-Based Approaches

Violence detection in video data is a challenging task due to the presence of complex patterns in the form of sequential information. For this purpose, numerous methods are developed, for instance, Chen et al. [18] used spatiotemporal interest points, including Harris corner detector, space–time interest points (STIP) [19], and motion scale-invariant feature transform (Mo SIFT) [7,20], for violence detection. Similarly, Lloyd et al. [21] developed new descriptors called grey level co-occurrence texture measures (GLCM), where changes in crowded texture are encoded by temporal summaries to detect violent and abnormal crowds. In addition, this, Fu et al. [22] developed a model to detect a fight scene; its function is to search a series of features based on motion analysis using three attributes, including motion acceleration, motion magnitude, and the motion region. These features are collectively called motion signal which is obtained by the summation of motion region. Similarly, Sudhakaran et al. [23] proposed a method where they used long short-term memory (LSTM) and the adjacent frame difference as an input into the model by encoding the changes that occur in the videos. Mahmoodi et al. [24] used a histogram of optical flow magnitude and orientation (HOMO) for violence detection. Recently, a violent activity recognition framework was presented by Fenil et al. [25] for a soccer game. They extracted histogram of oriented gradient (HoG) features from each frame. These features were used to train bidirectional long short-term memory (BD-LSTM) and ensure its usage for both forward and backward information access. This generated output contains information about violent scenes.

The approaches mentioned above tried to tackle many challenges in violence detection, including camera views, complex crowd patterns, and intensity variations. For instance, they failed to capture the discriminative and effective features by their extraction when variation occurs in the human body for violence detection. These variations occur due to viewpoint, significant mutual occlusion, and scale [26]. Next, [14] when considering ViF only, this method encounters a problem: If the flow vector for one pixel in two consecutive frames has the same magnitude and different direction, then the ViF’s effect is restricted because ViF detects no difference between these two flow vectors. Furthermore, earlier methods used flames, explosions, and blood for violence detection; these are limited because of low detection rates and can produce false alarms. Moreover, the HHMM based method [12] and HOMO [24] failed for complex crowd behavior recognition.

Recently, convolutional neural networks (CNNs) evolved to have higher accuracy and better results for various computer vision techniques, such as behavior recognition and security [10,27,28], object tracking and activity recognition [29,30], video summarization [31], and disaster management [8]. Inspired by the performance of CNNs in the mentioned domains, we tackle the problems mentioned above by proposing 3D CNN-based violence detection in surveillance. The key contributions of the proposed method are summarized in the following bullet points:

- Violence detection from video data is a challenging problem because of complex sequential visual patterns’ identification. The mainstream techniques use traditional low-level features for this task, which are inefficient at recognizing such complex patterns as well as being hard to implement in real-time surveillance. Considering the limitations of the existing techniques, we present a deep-learning-based 3D CNN model to learn complex sequential patterns to predict violence accurately.
- Most violence detection algorithms suffer from the problem of processing a massive number of unimportant frames, which results in occupying more memory and is very time-consuming. Considering this major limitation, we first detected the persons in the video stream using a pre-trained MobileNet CNN model. Only the sequence of 16 frames containing persons was passed to the 3D CNN model for final prediction, which helped achieve efficient processing.
- The current mainstream methods do not learn effective patterns due to lack of data in violence detection benchmark datasets and an often low accuracy rate. Inspired by the concept of transfer learning, the 3D-CNN was fine-tuned using publicly available benchmark datasets for violence detection in both indoor and outdoor surveillance. It experimentally dominates conventional hand-engineered features extraction algorithms by improving the accuracy rate.
- After obtaining the trained deep learning model, it was optimized using an OPENVINO toolkit to speed up and improve its performance at the model deployment stage. Using this strategy, the trained model was converted into an intermediate representation (IR) based on trained weights and topology.

The rest of the manuscript is organized as follows: Section 2 covers the proposed method, and the experimental evaluation is discussed in Section 3. A conclusion and future work are provided in Section 4.

## 2. Proposed Method

**Figure 1:**
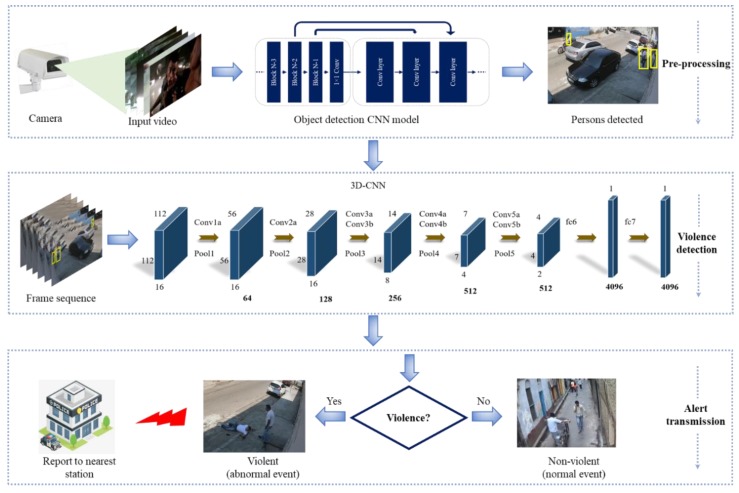
The framework of the proposed violent detection method. In the first phase, a video stream from a surveillance camera is acquired in which persons are detected. The second phase extracts deep features by feeding a selected sequence of frames to a 3D CNN model which detects the violent activity. Lastly, if a violent activity is detected, then we report this information to the nearest station to take immediate action before any injury or disaster occurs.

In this section, we discuss our proposed method in detail where a violent activity *Ă*_I_ is detected using an end-to-end deep learning framework. First, the camera captures the video stream V_I_, which is directly passed to a trained MobileNet CNN model to detect the people. When a person in the video stream is detected, the sequence Š of 16 frames is passed to the 3D CNN model for spatiotemporal features extraction. These features are fed to the Softmax classifier C_S_ to analyze the activity features at the end and give predictions. An alert is sent to the nearest security department when violence is detected so that they can take immediate action accordingly. The proposed method is further discussed in detail in the sub-sections, where each step is given in Figure 1. The e input and output parameters are described in Table 1 with symbols.

### 2.1. Pre-Processing

**Figure 2:**
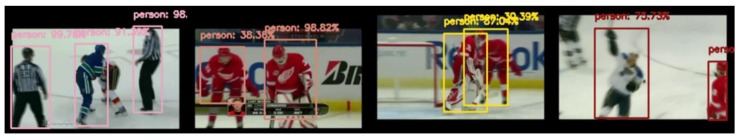
Persons detected in the frames of both classes from video clips of the hockey fight dataset using MobileNet-SSD.

Person detection is an essential step in our proposed method to ensure efficient processing before the violence detection step. In this section, we detect the persons in the video stream for efficient processing. Instead of processing the whole video stream, we process only those sequences that contain persons by avoiding unimportant frames. The video stream is fed into the MobileNet-SSD CNN model [32] for person detection. We used this CNN architecture because it helps the system to restrict for latency and size. MobileNet possesses depthwise separable convolutions to detect objects instead of regular convolutions. If depthwise and pointwise convolutions are counted separately, there are 28 layers, where every layer is followed by nonlinearity batch norm and ReLU except the final fully connected layer. The first convolutional layer contains a stride of two with a filter shape of 3 × 3 × 3 × 32 and has an input size of 224 × 224 × 3; its next depthwise convolution has one stride, the filter shape is 3 × 3 × 32, and the input size is 112 × 112 × 32. The MobileNet is mainly used for classification while its SSD version is used to locate the multibox detector, and their combination performs object detection. For this purpose, the SSD is added at the end of the network, which performs feedforward convolution and produces a fixed-size group of bounding boxes, to ensure the presence and detection of object instances in those boxes via extracting the features map and applying the convolution filters. The boundary box is composed of a predicted class with a probability for each class. The class with the highest probability indicates the object, while zero represents no object indication. A demonstration of person detection in some samples of the hockey fight dataset is shown in Figure 2.

### 2.2. Learning with 3D CNN

**Figure 3:**
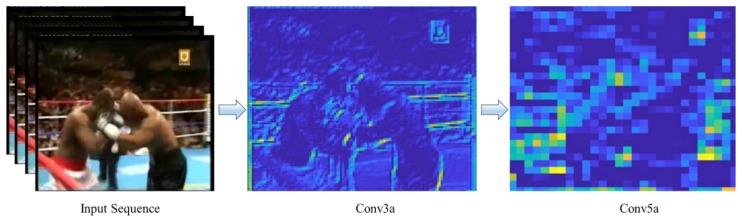
The input sequence is taken from violence in movies dataset. Feature map of the conv3a and conv5a is formed. As the process of the convolution proceeds, deeper features are extracted.

A 3D CNN is well-suited to extract spatiotemporal features and can preserve the temporal information better owing to its 3D convolution and pooling operation. In addition, in 2D CNNs, there is spatial information only, while a 3D CNN can capture all temporal information regarding the input sequence. Some of the existing methods use 2D ConvNets to extract the spatial correlation in video data, which possess temporal correlation. For instance, in [33,34], the 2D CNN processes multiple frames, and all the temporal feature information is collapsed. The 3D convolution operates by convolving a 3D mask on the cube designed via assembling attached frames. The obtained feature maps from the convolution layer are linked to multiple attached frames in the prior layer, capturing the motion information. Hence, the value on position *x*,*y*,*z* at the *q*th feature map in the *p*th layer with bias *t_pq_* is illustrated by

(1) $$N_{pq}^{xyz}=tanh(t_{pq}+\underset{k}{\sum }\overset{A_{p}-1}{\underset{a=0}{\sum }}\overset{B_{p}-1}{\underset{b=0}{\sum }}\overset{C_{p}-1}{\underset{c=0}{\sum }}w_{pqk}^{abc}N_{\left(p-1\right)k}^{\left(x+a\right)\left(y+b\right)\left(z+c\right)})$$

where *C_p_* is the 3D mask size with the temporal dimension and $w_{pqk}^{abc}$ is the (*a*, *b*, *c*)th value of the mask attached to the *k*th feature map in the prior layer. Only one type of feature is extracted by 3D convolutional mask from the frame cube since the weights of the kernel are replicated in the entire cube. In Figure 3, the feature maps of the 3D CNN obtained from two layers conv3a and conv5a are provided. The input sequence is taken from the violence category in the movies’ dataset. A principle for CNN is to increase the amount of feature maps in late layers by creating several kinds of features from the same feature maps. The input data to this network is a sequence of frames. Before starting the training process, the volume mean of training and testing data is calculated. The architecture of the network is fine-tuned to obtain these sequences as inputs. The final prediction at the Softmax layer is calculated as belonging to the violent or non-violent class.

### 2.3. Data Preparation and Usage

This section specifies the preparation of data and their usage for learning violence activity patterns. First, violence dataset Ď was used, containing Ň number of short video clips with different durations. Each video dataset contains two categories: i.e., violent class and non-violent class. Before the learning process, the whole dataset Ď was divided into a sequence of 16 frames Š with an 8-frame overlay between the two successive clips. Subsequently, having obtained the frames, we split the whole data into training and testing sets. For this purpose, we used 75% and 25% of data for training and testing, respectively. Once the training and testing data were obtained, we generated a file list containing the paths of training list Ĺ_Tr_ = {*S*_1_, *S*_17_, *S*_33_, …, *S_N_*} and testing list Ĺ_Te_ = {*S*_1_, *S*_17_, *S*_33_, …, *S_N_*}. The subscript of S is the starting frame number in the sequence where each path is given in the list, pointing towards the extracted frames in the directories.

### 2.4. C3D Network Architecture

**Figure 4:**
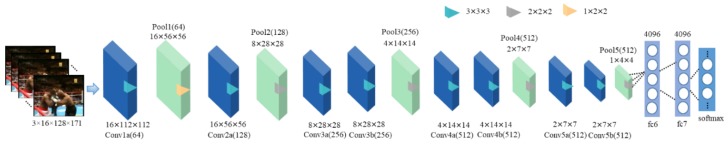
The architecture of C3D containing eight convolutional, five max pooling, and two fully connected layers, followed by SoftMax layer (Output). The three color cones represent the different size of filters for each layer.

Inspired by the performance of 3D CNN in [35,36,37,38], we also fine-tuned the 3D CNN model proposed in [36]. A starting version of the C3D model [36] was developed in 2014 with a version of Caffe [39]. This network consisted of eight convolutions: five pooling and two fully connected layers with a Softmax output layer. Each convolutional layer has 3 × 3 × 3 kernels with one stride, and all the pooling layers are max pooling with a 2 × 2 × 2 kernel size except for the first pooling layer where kernel size is 1 × 2 × 2 with two strides, preserving the time-based information. The number of filters in each convolution is 64, 128, 256, for first, second, and third layers, respectively. The kernels for each convolution have a defined temporal depth, with size D. The kernel size and padding used to apply the convolution were kept as 3 and 1, respectively. Two fully connected layers (fc6 and fc7) contained 4096 neurons and the Softmax layer containing N number of outputs depended on the classes of the dataset. In our case, the output is only two because we have only two classes: i.e., violent and non-violent scenes. The overall detailed architecture is illustrated in Figure 4.

This architecture of a 3D convolutional network obtained the short sequence of 16 frames as an input of size 128 × 171, but we used random crops of size 3 × 16 × 112 × 112 from the original input sequence at the time of training to avoid the overfitting problem and to achieve effective learning. After this, the sequence of frames is followed by 3D convolution and pooling operations. When training is performed, the network acts as a generic feature extractor. In fact, diverse features are learned at each layer of hierarchy in the network. The bottom’s activation layers contain smaller receptive fields making it sensitive towards patterns, such as corners, edges, and shapes, while the top activation layers contain larger receptive fields learning high-level and global features to collect complex invariances. Finally, the output label is predicted as violent or non-violent at the end.

### 2.5. Model Optimization

**Figure 5:**
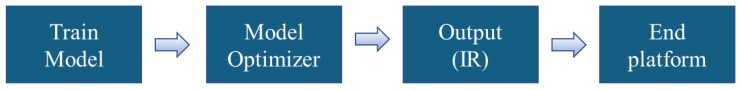
The flow of converting the trained model into intermediate representation (IR) format using model optimizer, where the IR format of the model is further used at the end platform.

Model optimization is the process used to generate an optimal and fine-tuned design model based on some prioritized constraints while keeping the model strength, efficiency, and reliability maximized. Optimizing the model enables CNN network inference at the end and speeds up the process by using pre-optimized kernels and functions. Inspired by these strategies, we used an open source toolkit known as OPENVINO provided by the Intel Corporation. This toolkit extends the work process across the hardware by maximizing its performance. It works on Intel hardware and takes pre-trained models, such as Caffe, ONNX, MXNet, and TensorFlow, as inputs and converts these into an IR using a model optimizer. The model optimizer is used to enable a transition between the training and deployment floor to adjust the model for optimal execution on the end platform. Figure 5 shows the flow and process of the model optimization, taking the trained model as input and producing an intermediate model. At the end platform, this output is deployed for further analysis.

## 3. Results

We conducted various experiments to evaluate the performance of the proposed method concerning three publicly available datasets for violence detection, such as violent crowd [14], hockey fight [7], and violence in movies [7]. To perform the experiments, we used different parameters and learning rates to achieve the greatest accuracy. Detailed descriptions of the datasets are given in Table 2. Furthermore, we compared our method with different handcrafted and deep-learning-based state-of-the-art methods to evaluate its accuracy and performance over three datasets. To perform the experiments, the Caffe toolbox was used to extract deep features on GeForce-Titan-X GPU. The operating system was Ubuntu 16.04 using Core^TM^ i5-6600 with 64GB RAM.

### 3.1. Datasets

This section describes the datasets used in the experiments. Each dataset has a different number of samples. A detailed explanation is given as follows:

#### 3.1.1. Violent Crowd

**Figure 6:**
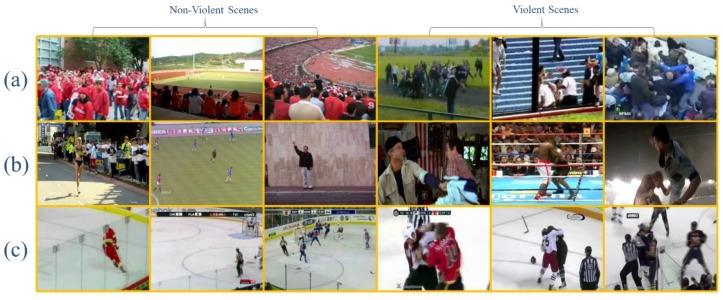
Sample video frames randomly selected from: (**a**) violent crowd [14], (**b**) violence in movies, [7] and (**c**) hockey fight [7].

The violent crowd dataset was presented by Hassner et al. [14]. This dataset contains 246 videos taken from YouTube, presenting different types of scenes and scenarios. At first, the dataset contains five sets of video clips. In each set, there are two categories: i.e., violent and non-violent. For the experiments, we merged these five sets to form two categories where 123 video clips are related to violent events, and 123 videos are related to non-violent clips. Each video clip has a resolution of 320 × 240 pixels with lengths varying from 50 to 150 frames. Some sample frames from this dataset are given in Figure 6.

#### 3.1.2. Violence in Movies

This dataset was introduced by Nievas et al. [7] for fight detection, and it consists of 200 videos clips, in which person-on-person fight videos have been taken from action movies while non-fight videos have been extracted from publicly available action recognition datasets. This dataset covers a variety of scenes, with an average resolution of 360 × 250 pixels and each clip is limited to 50 frames. In this dataset, a first person in the sequence has low or no camera motion. Some sample frames from this dataset are given in Figure 6.

#### 3.1.3. Hockey Fight

This dataset was introduced by Nievas et al. [7] and contains 1000 short video clips taken from the National Hockey League (NHL). In this dataset, 500 video clips are labeled as fight, and 500 are labeled as non-fight. Each clip consists of 50 frames with a resolution of 360 × 288 pixels. In the fight class, all the clips are related to fights in the hockey grounds, and the non-fight class is also related to the same environment containing non-fight clip so as to reliably detect violent scenes in sports videos. Some sample frames from this dataset are given in Figure 6.

### 3.2. Discussion

Table 3 explains the experiments performed on the violent crowd dataset, where the highest achieved accuracy was 98%, with 1.89 × 10^−9^ loss at the maximum iteration of 5000 with a base learning rate of 0.001. The loss value is given in scientific notation, which is equivalent to 1.89 × 10^−9^. We kept the learning rate normal because the learning rate has two terminologies for its usage. First, the learning rate should not be very large because it oscillates when searching for the minimal point and can cause drastic updates leading to divergent behaviors. Second, the learning rate should not be very small because it slows down the convergence towards the minimal point and requires too many updates before reaching the minimum point. At first, the learning rate is large, and the random weights at that position are far from the optimal point; then, it slowly and gradually decreases as further iterations proceed.

**Figure 7:**
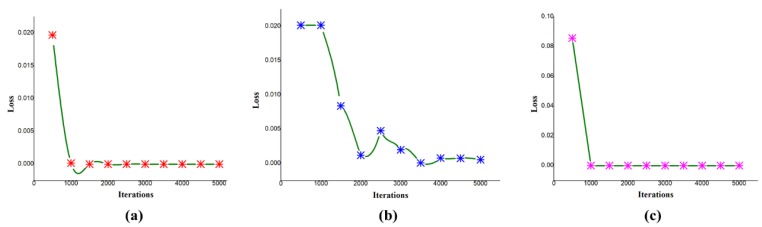
(**a**) Variation of loss with different iterations on hockey fight [7] dataset with a learning rate of 0.001; at the horizontal position, the initial iteration from zero grows towards the final iteration, which is 5000, while in the vertical a loss is given. The loss is decreasing as the iterations proceed; likewise in (**b**) the variation in loss with different iterations on violence in movies dataset [7] when the learning rate is 0.00001, it shows the loss is decreasing as the iterations proceed. (**c**) shows a variation of loss with different iterations on the violent crowd dataset [14], with a learning rate of 0.001, it shows that at the 500th iteration the loss is very high, but with further iterations, it decreases.

Table 4 explains the experiments performed on the violence in movies dataset [7], where the highest achieved accuracy was 99.9% with 1.67 × 10^−7^ loss at a maximum iteration of 5000 with the base learning rate of 0.001. After conducting experiments on the violence in movies dataset, we made various observations. For instance, detecting the fights in the movies dataset footage was easier than detecting it in the crowd dataset because when we tested the obtained model on the violent crowd dataset, we achieved 54% accuracy, which is low because fights in the violent crowd dataset are very varied in appearance or cinematography. In addition, the clips included a large number of people; however, in the violence in movies dataset, a majority of the videos clips contained person-to-person violence. Notwithstanding this, the hockey fight dataset was relatively very consistent. The same model was tested using the hockey fight dataset [7], in which the obtained accuracy was 63%, which is better than the accuracy obtained for the violent crowd dataset. We also tested the model obtained from the violent crowd on the other two datasets, i.e., violence in movies and hockey fight dataset, which gave an accuracy of 65% and 47%, respectively. The obtained accuracy on these two datasets is lower due to pattern footage because the hockey fight and violence in movies datasets contained person-to-person fights and the violent crowd dataset contained multiple numbers of persons. The graphical representation for the experiments performed in Table 4 is given in Figure 7.

Table 5 explains the experiment’s performance in relation to the hockey fight dataset [7], where the highest achieved accuracy was 96% with a 5.77 × 10^−4^ loss at the maximum iteration of 5000 and the base learning rate of 0.001. Furthermore, we evaluated the accuracy of the fine-tuned model of the hockey fight dataset [7] on the violent crowd dataset [14] and violence in movies, giving 52% and 49% accuracy, respectively.

In addition, we observed that changing the learning rate has an effect on loss and with iterations. In Figure 7a, the graph shows the change in loss with the variation in the number of iterations with a base learning rate of 0.001 for the hockey fight dataset. At the iteration of 500, the loss obtained is 1.97 × 10^−2^, which decreases as the number of iterations proceeds; at the maximum iteration of 5000, the obtained loss is 2.32 × 10^−7^ while keeping the same experiment, we only changed the learning rate to 0.0001, so the obtained loss at the initial iteration of 500 is 7.39 × 10^−2,^ and at the maximum iteration of 5000 the obtained loss is 5.77 × 10^−4^.

**Figure 8:**
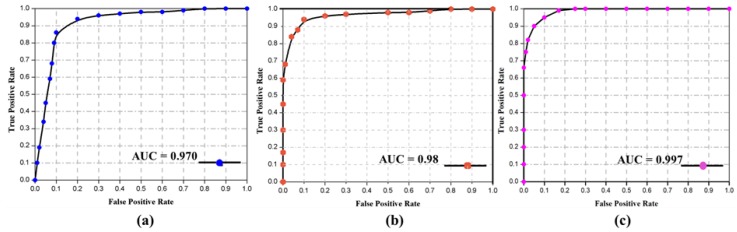
The ROC curve and comparison amongst the datasets based on AUC value, i.e., (**a**) Hockey fight dataset; (**b**) Violent crowd dataset; and (**c**) Violence in movies dataset.

The loss to iteration comparison for violent crowd is given in Figure 7c, where the loss decreases from the start and becomes less than zero after 1000 iterations. The loss for the violence in movies dataset in the initial stages is high; then, it decreases as iterations proceed. In this way, the loss obtained at the 5000th iteration becomes 5.4 × 10^−4^. The decrease in loss for the violence in movies dataset is graphically presented in Figure 7b, where the vertical axis represents the loss, and the horizontal axis represents the training iterations. We also evaluated the performance of the proposed method by examining precision, recall, and the comparison among the datasets by providing the values of area under the curve (AUC) in Table 6, which show the effectiveness of the proposed method on each dataset. In addition, the obtained confusion matrix is given in Table 7. The precision and recall values for each dataset ranges between X_min_, Y_min_ and X_max_, Y_max_, respectively. Here the X represents the precision, and Y represents recall for each dataset. The precision obtained for hockey fight, violence in movies, and violent crowd dataset is 0.9597, 1.0, and 0.9815, respectively, while the recall is 0.9667, 1.0, and 0.9876, respectively. We also calculate the time complexity of the proposed method, considering the testing phase during this experiment. For each 16 frame sequence, the average calculated time is 1.85 s, while, for a one-minute clip with 25 FPS it takes about 2 min and 54 s to complete the testing phase through all the sequences. We further evaluated the effectiveness of the proposed method by plotting the receiver operating characteristic (ROC) curve across the true positive rate and false positive rate. This is briefly illustrated in Figure 8, where the AUC values are compared for each dataset.

**Figure 9:**
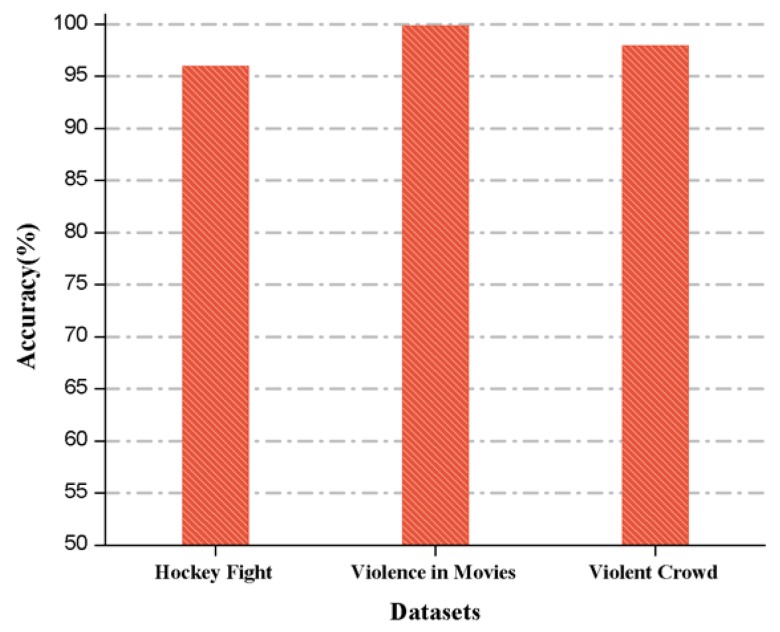
Comparative analysis of the proposed method on various datasets based on accuracy.

We also compared the accuracies for the benchmark datasets in Figure 9, where the highest achieved accuracy is 99.9% obtained in the movies dataset, 98% accuracy is obtained in the violent crowd dataset, and 96% is obtained in the hockey fight dataset.

### 3.3. Comparative Analysis

In this section, we compare the results of each dataset with existing state-of-the-art methods. The comparative analysis with all the state-of-the-art methods is shown in Table 8. In the first row, we present the results of method [17], which used oriented violent flows (OViF) for motion magnitude and AdaBoost as feature extraction, and SVM for classification. Using these parameters, they obtained an accuracy of 88% and 87.50% for the violent crowd and hockey fight datasets, respectively. Recently, another method [40] used Hough forests with 2D CNN to detect violence and obtained 99% accuracy on the violent movies dataset and 94.6% on the hockey fight dataset. Apart from this, there was another method [7] to detect violence in videos; this method used a spatiotemporal descriptor called space–time interest point (STIP), bag-of-words (BoW), and SVM to classify the output classes. They used only the violence in movies dataset and obtained 89.5% accuracy. Furthermore, we compared the results with another method [41], which used motion blobs and random forests for detection of the fast fight. They also used only the violence in movies dataset and obtained 96.9% accuracy. Moreover, in [42], two descriptors were used to detect and localize the abnormal behaviors; they used a simplified histogram of oriented tracklets (sHOT) combined with a dense optical flow to recognize abnormal behavior at the final result and obtained an accuracy of 82.2% for the violent crowd dataset. In [14], the authors used ViF and then classified the final prediction using SVM, where they used five-fold cross-validation for testing and obtained 82.90% accuracy for the hockey fight dataset and 81.3% for the violent crowd dataset. In method [43], the authors used the sliding window approach and improved the Fisher vector method to detect violence. They obtained accuracies of 99.5%, 96.4%, and 93.7% for violence in movies, violent crowd, and hockey fight datasets, respectively. Finally, in the last row, we present our approach, which obtained 99.9%, 98%, and 96% accuracies for violence in movies, violent crowd, and hockey fight datasets, respectively.

## 4. Conclusions and Future Work

In this paper, a three-staged end-to-end framework is proposed for violence detection in a surveillance video stream. In the first stage, persons are detected using an efficient CNN model to remove unwanted frames, which results in reducing the overall processing time. Next, frames sequences with persons are fed into a 3D CNN model trained on three benchmark datasets, where the spatiotemporal features are extracted and forwarded to the Softmax classifier for final predictions. Finally, an OPENVINO toolkit is used to optimize the model to speed up and increase its performance at the end platform. Experimental results over various benchmark datasets confirm that our method is the best fit for violence detection in surveillance and achieved better accuracy than several employed techniques. In the future, we intend to ensure our system is implemented over resource-constrained devices. Furthermore, we plan to propose edge intelligence for violence recognition work in the IoT using smart devices for quick responses.

## Figures and Tables

**Table 1:** Description of the input and output parameters used in the proposed method.

Symbols	Description	Symbols	Description
*Ă*_I_	Violent activity	Ď	Dataset
V_I_	Violent video	C_S_	Softmax classifier
Ƒ_N_	Number of frames	Fc	Fully connected layer
Š	Sequence of frames	Ň	Number of clips
Ĺ_Tr_	Training list	Ĺ_Te_	Testing list

**Table 2:** Detailed description and statistics of the used datasets.

Datasets	Samples	Resolution	Violent Scenes		Non-Violent Scenes	
			No. of Clips	Frame Rate	No. of Clips	Frame Rate
Violent Crowd [14]	246	320 × 240	123	25	123	25
Violence in Movies [7]	200	360 × 250	100	25	100	29.97
Hockey Fight [7]	1000	360 × 288	500	25	500	25

**Table 3:** Classification accuracies of the proposed method on the violent crowd dataset [14].

Learning Rate (Batch Size = 20)	Iterations	Loss	Accuracy
0.01	1000	1.30	55%
	3000	8.28 × 10^−1^	
	5000	7.07 × 10^−1^	
0.001	1000	1.52 × 10^−5^	98%
	3000	1.79 × 10^−8^	
	5000	1.89 × 10^−9^	
Testing the obtained model on violence in movies dataset [7]			65%
Testing the obtained model on hockey fight dataset [7]			47%

**Table 4:** Classification accuracies of the proposed method on violence in movies dataset [7].

Learning Rate (Batch Size = 20)	Iterations	Loss	Accuracy
0.001	1000	0	99.9%
	3000	0	
	5000	1.67 × 10^−7^	
1 × 10^−5^	1000	1.21 × 10^−2^	99.9%
	3000	1.99 × 10^−3^	
	5000	5.4 × 10^−4^	
Testing the obtained model on violent crowd dataset [14]			54%
Testing the obtained model on hockey fight dataset [7]			63%

**Table 5:** Classification accuracies of proposed method on hockey fight dataset [7].

Learning Rates (Batch Size = 20)	Iterations	Loss	Accuracy
0.001	1000	1.59 × 10^−4^	96%
	3000	0	
	5000	2.31 × 10^−7^	
0.0001	1000	9.1 × 10^−2^	96%
	3000	2.27 × 10^−6^	
	5000	5.77 × 10^−4^	
Testing the obtained model on violent crowd dataset [14]			52%
Testing the obtained model on violence in movies dataset [7]			49%

**Table 6:** Precision and recall with AUC values are compared for each dataset.

Datasets	Values				Precision	Recall	AUC
	TP	TN	FP	FN			
Hockey Fight [7]	262	230	11	9	0.95970696	0.966789668	0.970
Violence in Movies [7]	50	57	0	0	1.0	1.0	0.997
Violent Crowd [14]	160	128	3	2	0.981595092	0.987654321	0.98

**Table 7:** Confusion matrix for each dataset.

Classes\Datasets	Hockey Fight		Violence in Movies		Violent Crowd	
	Violent	Nonviolent	Violent	Nonviolent	Violent	Nonviolent
Violent	262	11	50	0	160	3
Non-violent	9	230	0	57	2	128

**Table 8:** Comparative analysis of the proposed method with state-of-the-art methods based on overall accuracy.

Methods	Datasets Accuracies (%)		
	Violence in Movies [7]	Violent Crowd [14]	Hockey Fight [7]
ViF, OViF, AdaBoost and SVM [17]	-	88	87.50
Hough Forests and 2D CNN [40]	99	-	94.6
STIP, BoW, and SVM [7]	89.5	-	-
Motion Blobs and Random Forests [41]	96.9	-	-
ViF [14]	-	81.3	82.90
sHOT [42]	-	82.2	-
Improved Fisher Vectors [43]	99.5	96.4	93.7
Proposed Method	99.9	98	96
